# SciELO 25 years: *The Scientific Electronic Library Online celebrates its 25^th^ anniversary*

**DOI:** 10.5935/0004-2749.2024-1007

**Published:** 2023-11-24

**Authors:** Eduardo M. Rocha, Tammy H. Osaki, Newton Kara Junior, Monica Alves, Claudia Moral

**Affiliations:** 1 Department of Ophthalmology, Otorhinolaryngology and Head & Neck Surgery Faculdade de Medicina de Ribeirão Preto, Universidade de São Paulo, Ribeirão Preto, SP, Brazil; 2 Department of Ophthalmology, Escola Paulista de Medicina, Universidade Federal de São Paulo, São Paulo, SP, Brazil; 3 Department of Ophthalmology & Otorhinolaryngology, Faculdade de Medicina, Universidade de São Paulo, São Paulo, SP, Brazil; 4 Department of Ophthalmology & Otorhinolaryngology, Faculdade de Ciências Médicas, Universidade Estadual de Campinas, Campinas, SP, Brazil; 5 Conselho Brasileiro de Oftalmologia, São Paulo, SP, Brazil

In September, the Scientific Electronic Library Online (SciELO) celebrated its 25^th^ anniversary with a fruitful international scientific event in Sao Paulo, Brazil. This time, the event was hybrid wherein even participants who could not attend in person had the chance to participate online. The acronym IDEIA was chosen to name the event, which stands for **I**mpact, **D**iversity, **E**quity, **I**nclusion, and **A**ccessibility. Furthermore, academics, editors, publishers, governmental agents, and different providers of services for scientific journals were present during the event to celebrate the conquests of SciELO and the future of science diffusion.

SciELO was created in 1997 as a joint project of the São Paulo Research Foundation and the Latin American and Caribbean Center on Health Sciences Information, which is also known by its original name the Regional Library of Medicine (BIREME, acronym in Portuguese), a specialized center of the Pan American Health Organization/World Health Organization for technical cooperation in scientific health information. The SciELO project was also supported by the Brazilian National Council for Scientific and Technological Development, and its major objectives at that time were to provide a platform to increase the visibility and impact of scientific research from Brazil and Latin America^(1)^.

In 2001, an editorial authored by Prof. Harley Edison Amaral Bicas celebrated the inclusion of Arquivos Brasileiros de Oftalmologia (ABO) in the SciELO collection. Significant progress and growth have been observed in the concept of “IDEIA” and the development of SciELO. Nowadays, SciELO provides its readership worldwide free, open-access to scientific journals from more than 16 countries, including Latin America, the Caribbean, Spain, Portugal, and South Africa, which is also called as Diamond open-access^(3)^. Furthermore, the SciELO collection provides access to over 1200 journals from various fields, such as health sciences, social sciences, and humanities.

Among the innovations announced in the 25^th^ anniversary meeting were the **SciELO Data**, a repository of raw data for articles published; **SciELO Books**, which is sponsored by university editorial offices with a hybrid collection of various ebooks; and **SciELO Preprints**, a repository where authors can make their papers available before journal acceptance and open for suggestions and reviews.

Another topic that has been discussed over the past few years and gained interest during the 25^th^ anniversary meeting of SciELO was the future of scientific publications in order to encourage the broad dissemination of knowledge. It was observed that financial sustainability is an important factor for most of the journals. Basically, all journals have limitations in material, manpower, and financial resources, while public funding agencies do not support the direct costs for the journals’ administration.

Faced with the growth of the open-access trend, commercial publishers have invested in launching and acquiring open-access journals, increasing the publication fees for authors as a way of compensating for the loss of profits from the sale of access to articles. It has been arguably suggested that research, in general, is financed by public resources, and peer review work is done free of charge by the scientific community. Imposing high fees for research publication and obtaining financial resources from public agencies are processes that involves taxpayers being double-charged to support any scientific endeavors. The alternatives proposed in the SciELO 25-year meeting were the Diamond Open Science and preprint publication, which are already offered by **SciELO and ABO**.

The richness of debates and interactions in the 25^th^ anniversary meeting predict a fruitful future for SciELO and the scientific journals and other platforms for science divulgation. Interesting comments on the 25-year anniversary meeting of SciELO and predictions for the 30^th^ anniversary of SciELO were published by Alberto Pelegrini Filho, suggesting that artificial intelligence (AI) and the regulation of the use of AI tools in scientific writing, as well as ethical issues related to its use, will gain major interest among the participants of the meeting in future^(4)^.

Owing to the partnership with SciELO, ABO has progressed to become a world-class scientific journal. As put by Prof. Bicas in his editorial celebrating the ABO SciELO partnership in 2001, “proud that we have arrived there, to stay”.
